# The effects of a people-centred model on longitudinality of care and utilization pattern of healthcare services—Brazilian evidence

**DOI:** 10.1093/heapol/czu077

**Published:** 2014-09-11

**Authors:** Ana Paula Scoleze Ferrer, Alexandra Valéria Maria Brentani, Ana Cecília Silveira Lins Sucupira, Ana Carolina Barsaglini Navega, Elisa Scanavini Cerqueira, Sandra Josefina Ferraz Ellero Grisi

**Affiliations:** Department of Pediatrics, School of Medicine, University of São Paulo, Av. Dr. Enéas de Carvalho Aguiar, 647., 05403-000 São Paulo, SP, Brazil

**Keywords:** Avoidable hospitalization, family medicine, healthcare evaluation, longitudinality, people-centred care, primary care, utilization of health services

## Abstract

Brazil is experiencing a time of change in pattern of care: from ‘traditional’ to Family Health Strategy (FHS), a model guided by the principles of people, family and community-centred medicine. The heterogeneity in care currently offered affects the primary care impact. This study aims to evaluate the longitudinality of care and correlate this primary care principle to the utilization pattern of care among patients hospitalized due to preventable conditions, comparing the two care models currently offered in Brazil. It is a cross-sectional, analytical and descriptive study with a quantitative approach. The sample consisted of 501 patients from 0 to 14 years old. Data was collected in 2011 and the *Primary Care Assessment Tool* (PCATool-Brazil) child version was used. Bivariate and multivariate analyses were performed including patient-related variables (age, maternal education, income and type of diagnosis) and care model. From the hospitalizations occurred during the period, 65.2% were Ambulatory Care Sensitive Conditions. Patients evaluated ‘longitudinality’ as regular. Both the care continuity dimension and the utilization pattern of care services showed a link with the care model offered. Findings suggest that the FHS care model, based on the assumptions of people-centred medicine, was associated with better ratings of care continuity, which was reflected in a more appropriate utilization pattern of care services.

**KEY MESSAGES**

- Lower rates of hospital admissions are described in patients with greater care continuity.
- Longitudinality is one of the healthcare characteristics strongly influenced by the assumptions of people-centred medicine.
- A people-centered model was associated with better ratings of care continuity, which was reflected in a more appropriate utilization pattern of care services.

## Introduction

From the need to expand access to healthcare in Brazil, the Family Health Programme was created in 1994, initially deployed in small municipalities. Since 2000, the programme has been considered, by the Ministry of Health as a reorientation strategy for the care model and its rapid spread began the conversion phase of the ‘traditional’ model, hitherto hegemonic (Sampaio *et al.* 2012). Family Health Strategy (FHS) can be defined as ‘a model of primary care, operated through preventive, promotional, recovery and rehabilitation strategies/actions, as well as palliative care provided by family health teams, committed to comprehensive healthcare, focused on the family unit and consistent with the socioeconomic, cultural and epidemiological community context’ (Andrade *et al.* 2006). In its design, the FHS team is multidisciplinary, consisting of a family doctor, a nurse, a nursing technician and four to six community health agents who work with clientele ascription, from the registration and tracking of population of a given coverage area. Community health agents have no formal training, they are recruited within the community where they live and thence, they play an important role by building the bridge between the community and the health care unit. They act through identifying and monitoring risk factors for individuals and the community during monthly home visits. Since they mandatorily live within the geographical coverage area of their health team, they know the environment and the community which they work with and thence, are recognized as leaders. They also promote health education and healthy behaviours during home visits (Brasil 2006). Thus, the FHS is a care model guided by the principles of people, family and community-centred medicine, providing primary care on an ongoing basis to individuals and families, regardless of age and the presence or absence of disease. In contrast to these FHS principles, the traditional model of primary care is characterized by a biomedical approach, provided by a medical specialist (paediatrician, general practitioner or obstetrician–gynaecologist), according to the population's spontaneous demand or by offering programmes based on epidemiological patterns of disease, vulnerability and risk (Morosini and Corbo 2007). Both models are provided by the public health sector and integrate the national Health System—Unified Health System (SUS in Portuguese).

The expansion of FHS, characterized by the rapid increase in population coverage and primary care offered to the population, was accompanied by positive impacts on health indicators such as lower child mortality rates (Macinko *et al.* 2006) and a decrease in the number of preventable hospitalizations among adults (Macinko *et al.* 2011; Mendonça *et al.* 2012). However, these results are not yet unanimous. FHS consolidation is difficult in big cities like São Paulo, the largest Latin American city. Urban dynamics, characterized by large social inequality, limiting conditions for access to healthcare system and a higher concentration of medium and high complexity services is considered as a ‘limiting’ factor to the full FHS expansion (Viana *et al.* 2008). Thus, Primary Healthcare (PHC) currently offered in São Paulo city is very heterogeneous, covering services based on ‘traditional’ model and FHS units. This heterogeneity in offered care affects PHC impact. An example is the persistence of high percentages of Ambulatory Care Sensitive Conditions (ACSC) in the city of São Paulo (Rehem and Egry 2011).

ACSC rates are one of the most widely used indicators today for evaluating PHC performance, as they describe that a high number of hospitalizations for these conditions can suggest problems in the access and/or quality of offered primary care (Casanova and Starfield 1995; Casanova *et al.* 1996; Caminal *et al.* 2004). Caminal and Casanova (2003) described a conceptual framework representing possible paths taken by patients within the health system, which may culminate in preventable hospitalization. The desired path is based on the assumption that PHC has the ability to solve 75–85% of the population's health problems and the small proportion that needs specialized care should be referred by primary care and returned to PHC as soon as the diagnostic and/or treatment procedure is completed. Therefore, for this it is essential that the PHC service is accessible, the population should identify it as a ‘gateway’ to the system and then receive the service in a timely manner. One of the attributes of PHC, that favours this desirable route, decreasing the ACSC, is longitudinality. Several authors have described lower rates of hospital admissions in patients with greater care continuity (Gill and Mainous 1998; Falik *et al.* 2001; Caminal *et al.* 2002; Menec *et al.* 2006). To Starfield (1998), longitudinality is a characteristic that refers to the continuity of care, which is essentially the established relationship, over time, between individuals and a professional and/or healthcare team and assumes the existence of a regular source of care and its use over time, regardless of a pathology existence. Therefore, it is one of the healthcare characteristics strongly influenced by the assumptions of People-Centred Medicine. The people-centred model of care, often confused with the patient-centred model, refers to the relationship that is established over time and that favours an accumulation of provider knowledge about the individual, facilitates the management that best suits their health needs and allows the patient's affiliation to the service (Starfield 2011).

Considering the high rates of ACSC, particularly in paediatric patients, and the coexistence of two different models of primary care in Brazil, this study aims to compare the two offered care models in relation to longitudinality care, from the users' perspective, and to correlate this finding to the utilization of PHC services among patients hospitalized due to preventable conditions.

## Methods

It is an analytical, descriptive and cross-sectional study, with a quantitative approach. This study is part of a larger study that assessed various aspects of PHC offered to children and adolescents in the western region of São Paulo, the largest Latin American city. This region has about 480 000 inhabitants, 94 000 between 0 and 14 years old and 44.5% of them exclusively use the public health system (Unified Health System—SUS in Portuguese). To serve this population, the PHC network consists of 14 basic health units (UBS in Portuguese) that attend people based on two care models: the traditional and the FHS model.

The sample comprised children and adolescents from 0 to 14 years old, admitted to the paediatric ward of the University Hospital of the University of São Paulo (Hospital Universitário da Universidade de São Paulo) (HU-USP) from 1 January to 31 December 2011. It was sought for a universal sample. Inclusion criteria were:

- Primary diagnosis on admission as an Ambulatory Care Sensitive Condition, according to the Brazilian list (Alfradique *et al.* 2009) and
- Child or adolescent being followed up at 1 of the 14 PHC units in the studied region. Exclusion criteria:
- The person in charge of the patient having already responded to the questionnaire in another hospitalization that occurred during the collection data period or;
- During the interview, child was not accompanied by a parent or person in charge;
- The person in charge of the patient had no knowledge about ambulatory follow-up or previous diseases or
- The person in charge of the child or adolescent disagreeing to respond to the survey after being informed about the terms of consent.

Three or four weekly visits to the HU-USP ward were performed throughout 2011, to collect data through interviews after considering inclusion and exclusion criteria. All interviewers received training on the instrument. Patients and care-givers were interviewed during the hospitalization period.

The instrument used for data collection was the Primary Care Assessment Tool validated in Brazil—Brazil PCATool—child version (Harzheim *et al.* 2006; Brasil 2010). This questionnaire was originally developed by Bárbara Starfield's team and colleagues at The Johns Hopkins Populations Care Policy Center for the Undeserved Populations (Cassady *et al.* 2000) and allows the evaluation of various attributes of PHC. In this study the questions regarding two domains of PHC were used, the longitudinality attribute being one of them as well as the questions related to pattern of healthcare services use (Appendix).

**Figure 1.**
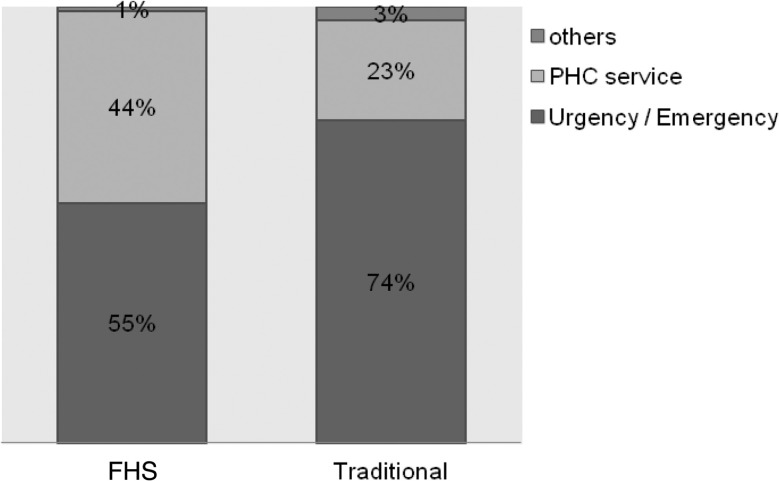
Type of service sought when a child (or adolescent) gets sick or needs orientations, according to the care model

**Figure 2.**
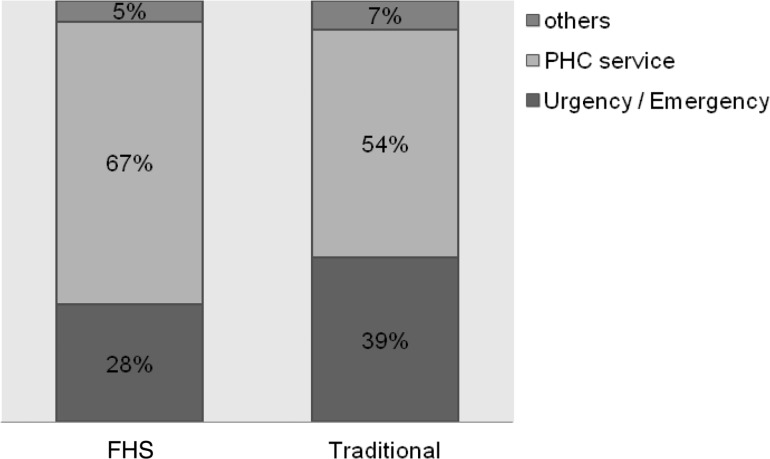
Type of service considered to be the one that knows the child (or adolescent) best as a person, according to the care model

**Figure 3.**
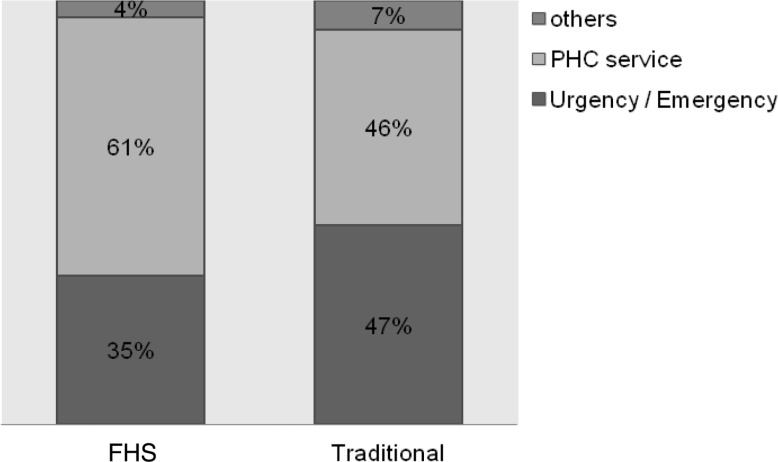
Type of service considered to be the most responsible for the child's (or adolescent’s) care according to the care model

(1) Longitudinality: this attribute consists of issues related to two aspects. The degree of affiliation, or how users recognize and identify themselves with the PHC service, and the bond established between the professional and the patient. The survey questions provided scores (0–10) for each subdomains according to the instrument’s instructions (Brasil 2010). The mean of the two scores (the degree of affiliation and the bond established between the professional and the patient) resulted in the longitudinality domain score (Tables 2 and 3). Scores lower than 6.6 were classified as inadequate in terms of longitudinality and values greater than or equal to 6.6 were considered suitable in terms of longitudinality for PHC principles. Initially, a bivariate analysis was performed, taking into account the proportion of each of these two groups (adequate or inadequate in terms of longitudinality) for each of the variables, using the Pearson's chi-square test. Studied variables were those related to the patient: sociodemographic (age, maternal education, family income) and diagnosis type (acute or chronic), and service related, that is to say, care model (traditional and FHS). The level of significance was *P* < 0.05. Subsequently, the variables that had a *P* > 0.20 were included in the multivariate analysis.
  Importantly, having adopted the Starfield design (1998), for which longitudinality refers to the interpersonal relationship between patient and provider, informational care continuity was not assessed in this study.
  Due to the relevance of the findings, the affiliation degree scores were presented separately (Figures 1–3). Regarding the degree of affiliation, the possible answers were: Yes or No, and when the answer was ‘yes’, they were asked which health service was considered for the answer. The analysis took into account the proportion of each of the service types responded (PHC services, emergency services and other) in relation to the care model (traditional and FHS).
(2) ‘Utilization Pattern of healthcare services’, which means how well PHC services are functioning as ‘gateway’ to the health system and consists of three questions.
  In the instrument, answers to these questions are presented as a Likert scale: 4. Surely yes; 3. Probably yes; 2. Probably not; 1. Surely not, and 9. Do not know/cannot remember. To perform the analysis, the answers were gathered into two groups Yes (answers 4 and 3) and No (answers 2 and 1) and ‘do not know/cannot remember’ answers were excluded. For each of the three questions, we compared the percentages of each response group (Yes/No) in relation to the care model received (traditional/FHS) by Pearson’s chi-square test, adopting a significance level of *P* < 0.05. The data underwent double typing and validation, using SPSS version 10.0 (SPSS Inc. Chicago) and Excel 2000 (Microsoft Corp. U.S.) software.
  Study was submitted for approbation by the Hospital’s Internal Review Board (CEP HU-USP) under registration no. 1039/10.

## Results

From a total of 2031 hospitalizations in the paediatric ward of the HU-USP from 1 January 2011 to 31 December, 1325 (65.2%) were ACSC. One hundred and eighty-eight patients were discharged before the interview and were considered lost, rendering 1137 patients. From these 636 met one or more exclusion criteria. The final sample consisted of 501 patients who met all inclusion criteria. Mothers were the main informants, answering 87% of the questionnaires.

From these 501 patients, 57.7% were male, with ages ranging from 20 days to 14 years (average 36.8 months) and 54.7% were white. Regarding socioeconomic data, 44.3% of mothers had eight years or less of schooling and 53.3% had a family income less than two minimum wages per month.

Hospitalization length ranged from 1 to 42 days, with an average of 4.9 days and respiratory diseases accounted for the vast majority of diagnoses at admission (78%). Regarding the type of ACSC, 410 (82%) cases were classified as acute and 91 (18%) as chronic.

Thirty-nine per cent of the sample was followed under FHS care. Table 1 shows the studied variables according to care model, the final composition of the sample was very similar between these two groups.

**Table 1.** Composition of the sample–variable distribution comparing the two care models

	FHS *n* (%)	Traditional *n* (%)
Age		
0–11 months and 29 days	83 (42)	132 (43)
1–4 years 11 months and 29 days	68 (35)	101 (33)
5–9 years 11 months and 29 days	35 (18)	43 (14)
10–14 years	11 (6)	28 (9)
Maternal education		
No education	1 (1)	7 (2)
Incomplete elementary	51 (26)	95 (31)
Complete elementary	24 (12)	45 (15)
Incomplete graduate	37 (19)	46 (15)
Complete school graduate	75 (38)	102 (34)
College	9 (5)	9 (3)
Family income		
Up to 1/2 minimum wage (MW)	5 (3)	8 (3)
From 1/2 to less than 1 MW	29 (15)	31 (10)
1 to less than 2 MW	74 (38)	120 (39)
2 to less than 5 MW	68 (35)	116 (38)
More than 5 MW	21 (11)	29 (10)
Character diagnosis		
Acute	160 (81)	250 (82)
Chronic	37 (19)	54 (18)

The score of the longitudinality attribute, calculated for the sample, was 4.93. Tables 2 and 3 show, respectively, the bivariate and multivariate longitudinality adequacy analyses, in relation to the studied variables.

**Table 2.** Bivariate analysis between the longitudinality adequacy and the patient and care model characteristics

Longitudinality			
	Inadequate *n* (%)	Adequate *n* (%)	*P* value
Age	347 (71)	145 (29)	0.1001
0–11 months and 29 days	139 (66)	72 (34)	
1–4 years 11 months and 29 days	119 (71)	49 (29)	
5–9 years 11 months and 29 days	62 (81)	15 (19)	
10–14 years	27 (75)	9 (25)	
Maternal education	344 (70)	145 (30)	0.7025
No education	7 (88)	1 (13)	
Incomplete elementary	104 (73)	38 (27)	
Complete elementary	43 (65)	23 (35)	
Incomplete graduate	55 (67)	27 (33)	
Complete school graduate	123 (71)	51 (29)	
College	12 (71)	5 (29)	
Family income	322 (71)	134 (29)	0.6898
Up to 1/2 minimum wage	9 (69)	4 (31)	
From 1/2 to less than 1 MW	37 (64)	21 (36)	
1 less to than 2 MW	142 (74)	51 (26)	
2 less to than 5 MW	126 (70)	54 (30)	
More than 5 MW	8 (67)	4 (33)	
Character diagnosis	345 (70)	145 (30)	0.9501
Acute	284 (70)	119 (30)	
Chronic	61 (70)	26 (30)	
Care model	347 (71)	145 (29)	0.0006*
FHS	120 (62)	75 (38)	
Traditional	227 (76)	70 (24)	

*statistically significance difference.

**Table 3.** Multivariate analysis between the longitudinality adequacy and the patient and care model characteristics

Longitudinality					
	Inadequate *n* (%)	Adequate *n* (%)	Prevalence ratio	Confidence interval	*P* value
Age group					
Under age 1	139 (40)	72 (50)	1.35	1.14–1.61	0.0832
1 year or older	208 (60)	73 (50)	1		
Diagnostic type					
Acute	284 (82)	119 (82)	1	0.90–1.42	0.5906
Chronic	61 (18)	26 (18)	1.13		
Care model					
Traditional	227 (65)	70 (48)	1	1.40–1.96	0.0024*
FHS	120 (35)	75 (52)	1.66		

*statistically significance difference.

Regarding the degree of affiliation, the majority (79.2%) answered ‘Yes’ when asked if there is a service to which they take the child or adolescent when sick or in need of some guidance about their health; 59.3% answered ‘Yes’ to the question whether there is any service that knows the child or adolescent better as a person, and 58.5% responded that there is a service that is more responsible for healthcare of the child or adolescent. The proportion of which service is considered in each of these issues, according to the care model received, is shown in Figures 1–3, respectively.

The answers to each of the three component issues ‘Utilization Pattern of Healthcare Services’ are shown in Table 4. These answers show that the sample’s vast majority (91%) uses PHC services for routine consultations and checking, without differences between the two care models. However, there is not the same utilization pattern when a new health problem rises, only 24% of respondents seeks PHC services in such times, being a statistically significant difference between the FHS model, whose users are more likely to use PHC, compared with the traditional model (question 2).

**Table 4.** Analysis of responses to each component question ‘Utilization Pattern of Healthcare Services’ according to the care model

	No *n* (%)	Yes *n* (%)	Total *n* (%)	*P* value
Question 1—When your child (or adolescent) needs a consultation/routine review, do you go to the PHC service before going to another service?				
FHS	14 (7)	183 (93)	197 (39)	0.3068
Traditional	31 (10)	273 (90)	304 (61)	
Total	45 (9)	456 (91)	501 (100)	
Question 2—When your child (or adolescent) has a new health problem, do you go to the PHC service before going to another service?				
FHS	132 (67)	65 (33)	197 (39)	0.0001*
Traditional	251 (83)	53 (17)	304 (61)	
Total	383 (76)	118 (24)	501 (100)	
Question 3—When your child (or adolescent) has to consult a medical specialist, or physician or PHC service, is it mandatory to forward it?				
FHS	68 (35)	129 (65)	197 (39)	0.7802
Traditional	100 (33)	204 (67)	304 (61)	
Total	168 (34)	333 (66)	501 (100)	

*statistically significance difference.

## Discussion

During the study period, of the 2031 hospitalizations that occurred at the paediatric ward of the HU, 65.2% were due to conditions considered sensitive to primary care. This ACSC ratio, compared with other studies conducted in Brazil, was high, making this research more relevant. Other Brazilian studies, in the same paediatric age group, found rates ranging from 35.6 to 57.6% (Lenz *et al.* 2008; Nedel *et al.* 2008; Caldeira *et al.* 2011). Possibly multiple causes are involved in explaining this high percentage of preventable hospitalizations. This study aimed to examine one of these factors—longitudinality care and its effects on the utilization pattern of PHC services, according to the care model.

The overall score achieved for continuity was 4.93, indicating that this attribute evaluation, from the users’ perspective, is only reasonable. This was an expected result, since the sample is composed of patients with negative results—a preventable hospitalization. The aim was to assess what were the variables that favoured better longitudinality of care and if it exerted some influence on the utilization pattern of healthcare.

Both bivariate analysis (Table 2) and multivariate analysis (Table 3) showed that no patient-related characteristics had an influence on the longitudinality. Only service characteristics, represented by the care model, were responsible for significant differences in the adequacy of this attribute. FHS users, a model driven by assumptions of people and family-centred medicine, were 66% more likely to consider the affiliation degree and bonding as appropriate. Considering that the survey questions only involve aspects relating to interpersonal longitudinality, findings reiterate the importance of the doctor–patient relationship favoured by a people-centred model, such as the practices described by Freeman and Hughes (2010), related to better care quality. Other authors, such as Roberge *et al.* (2001), from a qualitative evaluation, had reported that the contract between patient and professional relies on a strong interpersonal relationship. Our results support these guidelines, especially when we consider that, respectively, 67 and 61% of the FHS respondents consider that the PHC service is the one that knows the child best (Figure 2) and is most responsible (Figure 3) for taking care of their children.

We describe several positive impacts related to increased longitudinality. The continued attention allows us to establish a relationship of trust between the patient and the professional that assists the patient, improving adherence to proposed therapy and making them look for care earlier when needed. On the other hand, the professional who knows his patient better can make an earlier and more correct diagnosis, and the most appropriate treatments. Thus, the benefits of longitudinality are several: greater user-satisfaction, lower costs, better treatment and effectiveness, more preventive care, less need of care and lower hospitalization odds. These benefits are particularly evident in patients with chronic diseases (Starfield 1998; Freeman and Hughes 2010). This study aimed to analyse the relationship between longitudinality evaluation and the utilization patterns of healthcare services, once it had hospitalized patients with preventable conditions as study subjects.

In this sample, the analysis of the utilization pattern of PHC services revealed that population identifies PHC services as locations for routine care, but not as reference sites for resolving new or acute problems. This view was more common among traditional model users (Table 4). We found that, although more than half of the population, in both care models, identifies PHC service as the one that knows the child or adolescent best (Figure 2) and as most responsible for their care (Figure 3), there is a greater preference, especially among traditional model users, for seeking emergency care services during episodes of illness (Figure 1). This findings pattern shows that the use of PHC services in the region is not working properly as a ‘gateway’ to the system. As already described by the Caminal and Casanova framework (2003), this flow within the healthcare system is associated with higher chances of experiencing an avoidable hospitalization, since the physician who knows the child or adolescent best is not the same one that attends illness episodes. As discussed in relation to the observed results for the longitudinality verified score, one must consider that the sample consists of hospitalized individuals and, therefore, the chance of finding an inappropriate utilization pattern was greater. However, data helps to demonstrate that there was a positive influence of FHS in services utilization.

Service utilization depends on the active attitude of the user, but is strongly influenced by service characteristics. It appears that FHS model patients showed significantly higher PHC services demand during illness episodes (Table 4), with lower proportions in search of emergency and urgency services (Figure 1). This result possibly reflects the best assessment of longitudinality obtained from these patients. This positive association between continuity of care and the more appropriate use of health services, with less frequent visits to emergency services has been reported by other authors: Christakis *et al* (2001), who evaluated paediatric age group; Saultz and Lochner, in a comprehensive review published in 2005, and Hsiao and Boult (2008). Therefore, the result in this study gives higher recognition to PHC services as a regular source of care and as a ‘gateway’ to patients of the FHS model compared to others, possibly due to factors related to this model's principles: the assignment of clientele, the presence of community agents that allows greater user identification with the service and patient/family—centred approach, which strengthens the bond. The feeling of a ‘greater belonging’ to service contributes to its use, since, as quoted by Kovacs *et al.* (2005), the bond between the health team and patient has an impact on expectations in relation to meeting their needs.

Some aspects can be cited as study limitations. First, as the study population was of patients with avoidable hospitalization, the results are possibly worse compared to what would be observed in the general population; however, this does not invalidate the results since the objective was to compare the two care models. Likewise, the research design does not prove causality, only associations between variables related to patients, the care model, longitudinality and the utilization pattern of PHC services. The clinical management received by these children and adolescents was not verified neither the factors related to hospitalization policies, which also could have contributed to the hospitalization rate found. As the outcome studied was not the risk of ACSC occurring, these limitations did not interfere with the observed results.

## Conclusion

Results revealed that, from ACSC patients' perspective, continuity of care was assessed as just reasonable. However, it can be seen that the FHS care model, based on the people-centred medicine assumptions, was associated with better ratings of this aspect, which is reflected in a utilization pattern of the most appropriate service.

Although other authors had already described a positive association between longitudinality and the most appropriate use of services, the results of this study brought advances towards consistently correlating, the people-centred model—the best care longitudinality—and most adequate utilization pattern of care, since variables related to the patient were included in the analysis.

## Appendix

**Table:** Survey questions for each domain

1. Longitudinality
1.1 Degree of affiliation (A)
Answers: No or Yes (which health service was considered?)
A1) Where you take your child when (s)he gets sick or needs orientations about (her)his health? (Figure 1)
A2) What is the health service that knows your child best as a person? (Figure 2)
A3) What is the health service that you consider to be the one the most responsible for your child’s care? (Figure 3)
1.2 The bond established between the professional and the patient (D)
Answers: 4. Surely yes; 3. Probably yes; 2. Probably not; 1. Surely not, and 9. Do not know / cannot remember.
D1) Are you able to see the same provider (doctor, nurse) each time you takes your child to the PHC service?
D2) If you have a question about the health of your child, can you call and speak with the provider (doctor, nurse) who knows your child better?
D3) Do you think that the provider (doctor, nurse) understand what you say and your questions?
D4) Does the provider (doctor, nurse) explain things clearly?
D5) Does the provider (doctor, nurse) give you enough time to talk about your worries or problems?
D6) Does you feel comfortable telling your worries and your child’s problems to the provider (doctor, nurse)?
D7) Does the provider (doctor, nurse) know your child more as a person than just as someone with a health problem?
D8) Does the provider (doctor, nurse) know the complete medical history of your child?
D9) Does the provider (doctor, nurse) know which medications your child use?
D10) Would the provider (doctor, nurse) meet your family if you consider necessary to your child?
D11) Would you change the health service to another service if this were easy to do?
2. Utilization pattern of healthcare services
Answers: 4. Surely yes; 3. Probably yes; 2. Probably not; 1. Surely not, and 9. Do not know/cannot remember.
B1) When your child (or adolescent) needs a consultation / routine review, do you go to the PHC service before going to another service?
B2) When your child (or adolescent) has a new health problem, do you go to the PHC service before going to another service?
B3) When your child (or adolescent) needs a consultation with a specialist. do you need a referral from the PHC service?
